# Crowdsourcing Knowledge Production of COVID-19 Information on Japanese Wikipedia in the Face of Uncertainty: Empirical Analysis

**DOI:** 10.2196/45024

**Published:** 2023-06-29

**Authors:** Kunhao Yang, Mikihito Tanaka

**Affiliations:** 1 Graduate School of Sciences and Technology for Innovation Yamaguchi University Ube Japan; 2 Faculty of Political Science and Economics Waseda University Tokyo Japan

**Keywords:** scientific uncertainty, COVID-19, Wikipedia, crowdsourcing information production

## Abstract

**Background:**

A worldwide overabundance of information comprising misinformation, rumors, and propaganda concerning COVID-19 has been observed in addition to the pandemic. By addressing this data confusion, Wikipedia has become an important source of information.

**Objective:**

This study aimed to investigate how the editors of Wikipedia have handled COVID-19–related information. Specifically, it focused on 2 questions: What were the knowledge preferences of the editors who participated in producing COVID-19–related information? and How did editors with different knowledge preferences collaborate?

**Methods:**

This study used a large-scale data set, including >2 million edits in the histories of 1857 editors who edited 133 articles related to COVID-19 on Japanese Wikipedia. Machine learning methods, including graph neural network methods, Bayesian inference, and Granger causality analysis, were used to establish the editors’ topic proclivity and collaboration patterns.

**Results:**

Overall, 3 trends were observed. Two groups of editors were involved in the production of information on COVID-19. One group had a strong preference for sociopolitical topics (*social-political* group), and the other group strongly preferred scientific and medical topics (*scientific-medical* group). The *social-political* group played a central role (contributing 16,544,495/23,485,683, 70.04% of bits of content and 57,969/76,673, 75.61% of the references) in the information production part of the COVID-19 articles on Wikipedia, whereas the *scientific-medical* group played only a secondary role. The severity of the pandemic in Japan activated the editing behaviors of the *social-political* group, leading them to contribute more to COVID-19 information production on Wikipedia while simultaneously deactivating the editing behaviors of the *scientific-medical* group, resulting in their less contribution to COVID-19 information production on Wikipedia (Pearson correlation coefficient=0.231; *P*<.001).

**Conclusions:**

The results of this study showed that lay experts (ie, Wikipedia editors) in the fields of science and medicine tended to remain silent when facing high scientific uncertainty related to the pandemic. Considering the high quality of the COVID-19–related articles on Japanese Wikipedia, this research also suggested that the sidelining of the science and medicine editors in discussions is not necessarily a problem. Instead, the social and political context of the issues with high scientific uncertainty is more important than the scientific discussions that support accuracy.

## Introduction

### Background

In addition to the global COVID-19 pandemic since 2020, a worldwide eruption of an overabundance of information, including misinformation, rumors, and propaganda about COVID-19 has been observed [1-3]. The World Health Organization (WHO) referred to this as an “infodemic” [4,5]. Many studies [2-9] have noted that compared with the threat of the virus itself, the infodemic of COVID-19 has led to an even more significant impact on the world. The infodemic, which spread through social media and caused fear and panic throughout the global population, was closely related to the stigma and hatred for those from the region or country where the disease was believed to have originated.

Indeed, over the past 3 years, the world has faced the challenge of scientific uncertainty related to COVID-19 owing to a lack of scientific knowledge and empirical data about the virus. However, labeling the situation pathologically as an infodemic, as if it were a linguistic disease, and attempting to contain it through inoculations of *correct* information is at odds with the recommendations of experts involved in the practice and study of scientific or risk communication [10-12]. Such paternalistic solutions go against the democratic ideals of our society and science per se. The actual problem could be the chaos stemming from the struggle of an information society exposed to an unknown risk. The way to address this issue was not found in the situation itself but should rely on the best practices of democratic knowledge coproduction that continue to function even during such turmoil [13]. Therefore, we turned to Wikipedia’s editing processes.

To address the challenges of discussing facts during the scientific uncertainty surrounding COVID-19, Wikipedia, a crowdsourced web encyclopedia, has become an important reference [14-18]. According to a report from the Wikimedia Foundation [14], a total of 6950 Wikipedia articles related to COVID-19 in 188 languages had already been created before December 8, 2020. These articles received >500 million page views from readers around the world. More importantly, it was also found that COVID-19–related information on Wikipedia was of higher quality and reliability than other sources, such as Twitter, Facebook, and even preprinted research papers [18-20]. This was because most COVID-19–related information (ie, articles) on Wikipedia was directly sourced from credible media (eg, academic journals with peer review) or official institutions (eg, the WHO) [18-20]. These sources ensured the high quality and reliability of the information on Wikipedia in the face of the COVID-19 information turmoil. Moreover, 1 of the authors (MT) has been a member of a science advisory board focusing on COVID-19 since 2020. As an expert who has followed the latest scientific information on COVID-19 for these 3 years, it is the opinion of this author (MT), albeit subjective, that articles on Wikipedia were largely kept fair and accurate throughout this period. Nevertheless, despite the high quality of the information, many previous studies [15-17,21] have also noted that most of Wikipedia’s editors (ie, the users who edit the articles) can be said at best to be amateurs (without professional knowledge on the topics they edit). Thus, it is vital to understand how Wikipedia editors produced reliable information on COVID-19, especially considering that they are not experts in the relevant fields.

### Objectives

To identify how the editors of Japanese Wikipedia produced COVID-19 information, this study investigated collaborative patterns among these editors. Specifically, this study focused on the following 2 questions:

1. What knowledge and preferences did the Wikipedia editors who participated in producing COVID-19 information have? Previous studies [21-25] have found that collaboration among editors with different preferences is a key to creating high-quality information on Wikipedia. However, recent studies [26-28] have also found that on COVID-19 topics, collaboration among editors with diverse preferences can foment conflict and make collaboration counterproductive. This is because the topics (eg, the source of the virus and the risks of vaccinations) relating to COVID-19 are so controversial that editors with different preferences can easily hold different opinions and may be unable to reach a consensus. Thus, by investigating the preferences of the relevant editors, this study contributes to understanding how the Wikipedia community addressed the paradox between diversity and consensus during information production on controversial topics related to COVID-19.
2. What type of preference characterized the editors who played leading roles (ie, made more contributions) in the Wikipedia COVID-19 information production process? Owing to the large amount of information on COVID-19, the type of information that should be prioritized during information processing is a core question for containing the information turmoil related to COVID-19 [2-6,8,9]. Previous research [29] found that editors with different knowledge and preferences deployed different amounts of attention as members of the Wikipedia community to various topics. Thus, the preferences of the editors who played a prominent role were prioritized in the COVID-19 information overload. Therefore, by investigating the different roles played by editors with distinct preferences in producing information for COVID-19 topics on Wikipedia, this study indicates the types of COVID-19 information that were prioritized for publication.

The epidemic curves of the pandemic varied in each country, where different policies and social responses were observed. Therefore, this analysis was conducted only concerning Japanese Wikipedia. This was done to minimize and simplify the environmental effects of the COVID-19 epidemic by analyzing cases where the political and social scope of the nation coincides with the language used.

To investigate the research questions, the remaining parts of this study empirically analyze a large-scale data set of editing histories of Wikipedia editors using machine learning methods. This study provides a deeper understanding of collaboration patterns in Wikipedia’s COVID-19 information production process. In addition, these results contribute to developing procedures for designing media architecture to support democratic debates, which can counteract or contain uncertainty.

## Methods

### Overview of the Wikipedia Data Set

This study used a large-scale data set collected from Japanese Wikipedia. This data set was selected for 2 reasons. First, unlike some languages, nearly all Japanese Wikipedia editors reside in the same country [14]. This allowed us to determine how editors responded to the changing circumstances of the pandemic in a particular country regarding the specific policies in this country (eg, the mobility restrictions imposed in Japan). Second, according to a report by the Wikimedia Foundation [14], the COVID-19 information found on Japanese Wikipedia received an exceptionally high level of interest (eg, >12 million page views and 31,910 edits) relative to Wikipedia articles in other languages that are mainly spoken in a single country (eg, Dutch and Korean). We have verified this high level of interest and also showed that COVID-19–related articles in Japanese Wikipedia have significantly high quality (Multimedia Appendix 1 [30,31]). This indicates that Wikipedia is a prominent information source for COVID-19 in Japan. For these reasons, the Japanese Wikipedia was used as the data source to understand the production of COVID-19 information on Wikipedia.

On the basis of a widely used algorithm in previous studies [29,32], a total of 133 Japanese Wikipedia articles strongly related to COVID-19 were collected. A total of 1857 editors (referred to as the *focal editors* hereafter; 533 anonymous editors and unregistered editors were excluded; Multimedia Appendix 1) were involved in the 31,910 edits of these articles from February 6, 2020 (ie, the date when the first COVID-19–related article was created in Japanese Wikipedia) to February 12, 2022 (ie, the date when the data were collected). To identify the preferences of these focal editors, we collected their editing histories, which recorded what the focal editors had edited elsewhere on Wikipedia. Previous studies [15-18] have found that because the editing histories reflect the genre of topics the focal editors mainly focused on, this could be a means of interpreting an editor’s preference. The focal editors’ histories included 2,015,109 Wikipedia pages (including talk pages and discussion pages), of which 108,628 (5.39%) were articles (including templates and temporary articles). In the following analysis, we mainly focused on these 108,628 articles. In addition to the contents of these Wikipedia pages, the citation relationships among the obtained articles were collected. Thus, when a Wikipedia article cites another Wikipedia article in its content, a hyperlink is added to its content to assist the readers in verifying the cited article. These hyperlinks among the articles were also recorded.

### Dividing Editors With Different Knowledge Preferences Into Different Groups

First, a clustering analysis was conducted to divide focal editors into groups according to their editing histories. This analysis was designed to group editors with editing histories that focused on similar topics together. Thus, the groups can be considered to contain editors sharing the same preference. To perform this clustering analysis, a Wikipedia article network was constructed using hyperlinks among the Wikipedia articles featured in the editing histories of the focal editors. Here, the nodes represent Wikipedia articles, and the connections between them represent hyperlinks among them. As noted, because the hyperlinks only exist among articles that mention each other, connected nodes are considered to have similar topics. Thus, the distance between 2 nodes reflects the proximity of the issues of the article [33].

Using the graph neural network method node2vec [34,35], the nodes in the network were embedded in a 128-size vector. Node2vec is an extension of word2vec. It uses a shallow neural network to learn vectors representing the network nodes. According to previous research [34,35], the cosine distance among the vectors effectively reflects the distance among the nodes in the network. As articles with related topics have smaller distances between them in the network, the cosine distance among nodes reflects the similarity of their topics.

Next, the focal editors were represented by summing the vectors of all the articles edited by a given focal editor. This sum generally represents the main topics of the editor’s editing history [36], and the cosine distance between the 2 vectors indicates the similarity between the editing histories of the 2 focal editors represented [36].

Finally, according to the cosine distance among the vectors of the focal editors, the focal editors were clustered into groups. It is worth noting that the cosine distance indicates the angle between 2 vectors, regardless of their lengths. Therefore, this method allowed us to identify the difference in the topics of editors’ editing histories reflected by the angle between vectors instead of their activities reflected by the length of the vectors. To determine how many groups the editors should be clustered into, hierarchical clustering was used to create a dendrogram of the focal editors. This dendrogram encompasses all possible ways in which focal editors can be clustered, ranging from treating each editor as an individual cluster to consolidating all editors into a single cluster. From the dendrogram, the Schwartz Bayesian information criterion (BIC) was used to select the optimal number of groups for clustering the editors [37]. Following previous research [37], if the number of groups changes, the BIC of the clustering model also changes. Therefore, the optimal number of groups is that which achieves the smallest BIC.

**Figure 1:**
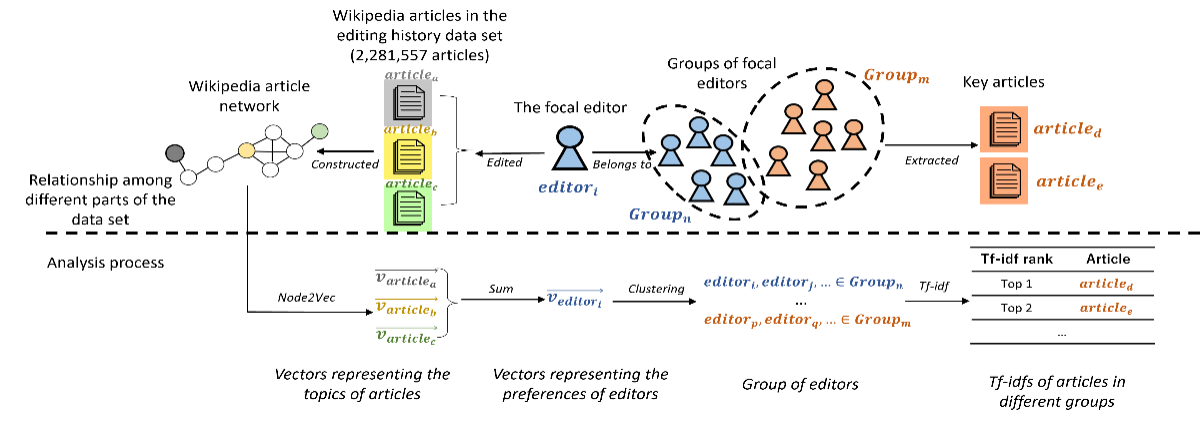
The entire process of identifying editors’ knowledge preferences. The upper half of the figure shows the relationships among the parts of the data set (Wikipedia article network, editing history data, focal editors, and key articles). The figure’s lower half shows the analysis process from creating a network of Wikipedia articles to identifying the preference of different groups of editors. TF-IDF: term frequency–inverse document frequency.

Using the abovementioned computations, focal editors were clustered into groups based on preference. These computations are illustrated in Figure 1.

### Identify the Specific Knowledge Preferences of the Editors in Different Groups

Using the clustering results, the specific shared preferences of the focal editors in each group were identified by extracting key articles from the editing histories of the groups. These key articles were defined as those commonly edited by one group but seldom edited by other groups. The key articles were identified by calculating the term frequency–inverse document frequency (*Tf-idf*) for each based on the editing histories of the groups [38]. For example, for articles *a* and *A*, as the set of articles in the editing histories of the focal editors in group *g*, the *Tf-idf* of *a* for *g* equals to the multiple of two values: (1) the ratio between the number of times article *a* was edited by the editors in group *g* and the total number of edits made by the editors in group *g* and (2) the logarithm of the ratio between the total number of the groups and the number of groups whose editors edited article *a*. Given the editing history of the editors in group *g*, articles with large *Tf-idf*s (the top 10 *Tf-idf*s) were considered the key articles for group *g*. These articles reflect the main differences in the editing histories of the editors in group *g* relative to those in other groups [38]. The topics of the key articles of group *g* can then represent the editors’ preferences in group *g*. These computations are illustrated in Figure 1.

The categories of the articles were investigated to further indicate how the topics of the key articles of group *g* could represent the knowledge preferences of the editors in group *g*. Wikipedia defines the category of an article to identify the type of topics covered by an article. This property identified the categories of the articles in group *g*’s editing histories. Specifically, the percentage of articles in group *g*’s editing history whose categories were related to the topics of group *g*’s set of key articles was calculated. A high percentage indicated that the topics of the key articles of group *g* effectively represent the preferences of the focal editors in group *g*.

The abovementioned analyses identified the specific knowledge preferences of the editors in different groups.

### Static Analyses of the Contributions Made by Different Groups of Editors in Terms of COVID-19 Information Production on Wikipedia

Next, we investigated the extent to which sets of editors grouped by their preferences contributed to the production of COVID-19–related information on Wikipedia. In particular, the group that played the most prominent role in editing COVID-19 articles on Wikipedia was investigated.

This was calculated by means of a static approach to the dynamic editing process of articles related to COVID-19 on Wikipedia. Specifically, 4 indicators were computed to reflect the contributions of the editors of group *g*. First, the percentage of focal editors in group *g* relative to the total number of focal editors was calculated. Next, the number of edits made by the editors of group *g* to the articles on COVID-19 was computed.

Furthermore, considering that some edits may involve significant changes to an article, whereas others may only entail minor modifications, the extent of change by an editor in group *g* was investigated. In particular, we calculated the size of the change, measured in bits, resulting from the edits made by the editors in group *g*. Finally, the number of references in a Wikipedia article reflected its reliability [18]. Therefore, the number of added references was obtained to measure the contributions made by group *g*’s editors to an article’s reliability.

### Dynamic Analyses of the Contributions Made by Different Groups of Editors in the Production of COVID-19–Related Information on Wikipedia

Analyses of static and dynamic factors were conducted to investigate which group of editors played the leading role in editing the COVID-19 articles on Wikipedia. In addition, the change in the focal editors’ contributions across time was considered. In particular, these dynamic analyses were used when a group of focal editors contributed more to editing COVID-19 articles. The focus was on the relationship between changes in focal editors’ contributions and the number of new COVID-19 cases detected in Japan over time. Therefore, given the identities of the focal editors in group *g*, the tendency of their contributions in editing COVID-19 articles relative to the number of new COVID-19 cases in Japan was analyzed.

**Figure 2:**
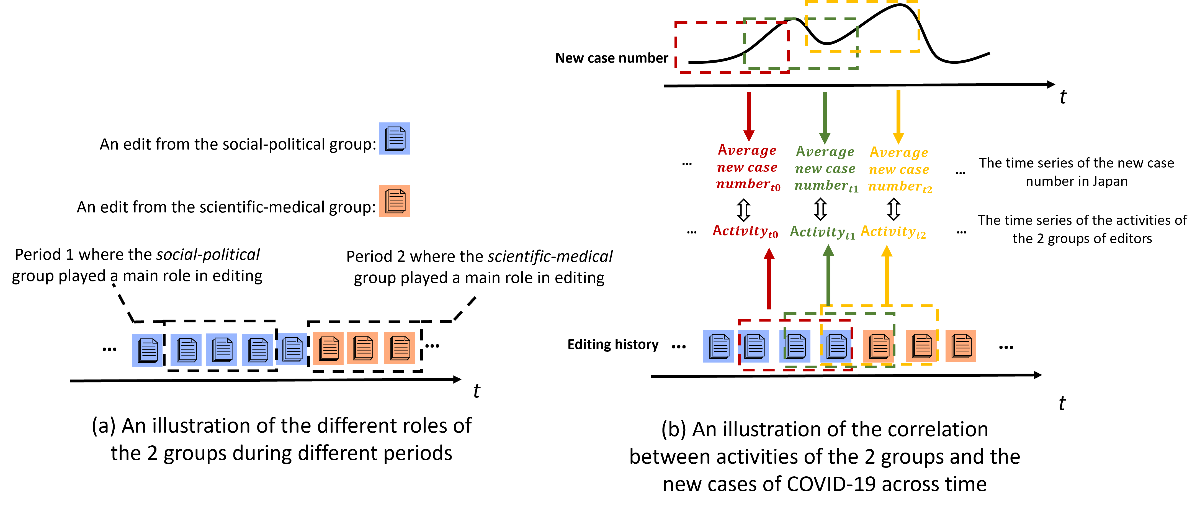
An illustration of the computation of the case variable, the contribution variable, and the Pearson correlation between the 2 time-series variables. The upper part presents a hypothetical case of the computation of the case variable. The lower part presents a hypothetical case of the computation of group g’s contribution variable. Colored rectangles represent the moving time windows used in the computation.

Two time-series variables were computed to perform dynamic analyses: the case variable reflects dynamic changes in the number of new COVID-19 cases in Japan over time, and the contribution variable reflects the dynamic changes in the contributions made by a particular group of focal editors across time. First, the case variable was computed using the public data set of new COVID-19 cases published by the Ministry of Health, Labour and Welfare of Japan [39]. Given a particular period *d*, represents the average number of new cases per day during this period. Then, the time series of the case variable (..., *case_d−1_*, *case_d_*, *case_d+1_*, ...) was generated using a moving time window (eg, 7 days) to change the period *d* day by day (Figure 2).

The contribution variable was computed using a Bayesian model, which inferred the contribution variable according to the times of edits made by the editors in a given group. More specifically, given that *N* represents the times of all edits made by all focal editors on the set of articles related to COVID-19 during period *d* and *M* (where the size of *M* is always less than that of *N*) represents the times of edits implemented by the editors in group *g* during period *d*, according to Bayesian rules, the contribution variable of the editors in group *g* was then defined in the following distribution as *Beta*(1 + *M*, 1 + *N* – *M*) [40]. The value of group *g*’s contribution variable during period *d* 
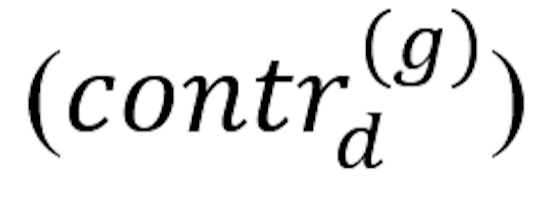
 was then defined as the mean of the distribution, equal to 
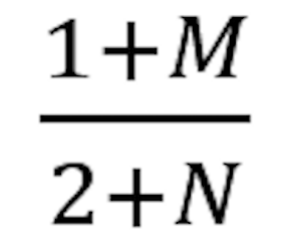
. Finally, as with the computation of the case variable, the time series of the contribution variable 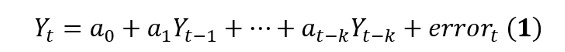 was generated using a moving time window, wherein period *d* changes day by day (Figure 2).

In summary, larger (smaller) values of the contribution variable indicate more (fewer) contributions by focal editors in group *g* to the production of COVID-19 information on Wikipedia during period *d*. In addition, as the values of the variable range from 0 to 1, values >0.5 reflect that the focal editors in group *g* played the primary role in editing COVID-19–related articles during period *d*. The contribution variable is computed in such a way as to down-weight the small-sample effects in *N* [40]. Taking into account cases where *N* equals 1, this indicates that all COVID-19 articles were edited only once during *d*. In this case, the percentage of edits by group *g* is equal to either 1 (ie, fully contributed by the editors in this group) or 0 (no contributions). However, group *g*’s contribution variable will be either 1/3 or 2/3. Thus, less extreme values will appear in group *g*’s contribution variable [40].

Finally, the Pearson correlation between the case and contribution variables was calculated for group *g*. A positive (negative) correlation coefficient indicates that the editors in group *g* tended to make more (fewer) contributions to information about COVID-19 when more COVID-19 cases were detected in Japan. The Granger causality test was used to determine whether the editors’ editing behaviors were causally affected by the number of new cases [41]. The Granger causality test is a statistical method used to investigate causal relationships between time-series variables. Mathematically, given 2 time-series variables *X_t_* and *Y_t_* at time lag *k,* the Granger causality test compares the following 2 autoregression models:



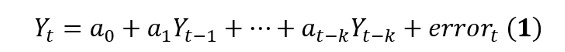





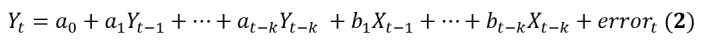



If the model 2 predicts *Y_t_* significantly better than model 1, Granger causality is identified between the variables *X_t_* and *Y_t_*. In our case, this would indicate that dynamic changes in the number of new cases in Japan caused dynamic changes in editors’ editing behaviors. In this study, lag *k* was set to 2.

### Data and Code Accessibility

The raw data and the Python code created for the statistical analyses are available in a dedicated Open Science Framework repository [42]. More details about the repository can be found in Multimedia Appendix 1.

## Results

### Collaboration Between Focal Editors With a Sociopolitical Preference and Editors With a Scientific-Medical Preference

Using the clustering method described in the *Methods* section, the focal editors were divided into 2 groups based on their editing histories. The first group comprised 1546 editors, and the second group comprised 311 editors.

Table 1 presents the key articles with the top 10 *Tf-idf* values of the 2 groups. The first (second) row shows the articles that were edited more often by the first (second) group but seldom by the second (first) group. The main difference between the knowledge preferences of the 2 groups of focal editors was that the first group tended to focus on editing articles on *social* and *political* topics, whereas the second group focused on *scientific* and *medical* topics. From these results, it was concluded that the first group consisted of editors with a strong knowledge preference for sociopolitical issues (the *social-political group*). In contrast, the second group strongly preferred science and medicine topics (the *scientific-medical group*).

**Table 1:** Titles of different key articles for the 2 groups of focal editors. The Japanese titles are given in parentheses.

Rank	Social-Political Group	Scientific-Medical Group
Top 1	“The Republic of Korea” (“大韓民国”)	“Prevalence of coronavirus infections in Japan in 2019” (“日本における2019年コロナウイルス感染症の流行状況”)
Top 2	“United States of America” (“アメリカ合衆国”)	“Global epidemic of new coronavirus infections (2019-)” (“新型コロナウイルス感染症の世界的流行 (2019年-)”)
Top 3	“Democratic People’s Republic of Korea” (“朝鮮民主主義人民共和国”)	“SARS coronavirus 2“(“SARSコロナウイルス2”)
Top 4	“People’s Republic of China” (“中華人民共和国”)	“Coronavirus infection prevalence status in 2019 by country/region” (“国・地域毎の2019年コロナウイルス感染症流行状況”)
Top 5	“Emperor Showa” (“昭和天皇”)	“Template: 2019-nCoV “(“Template: 2019-nCoV”)
Top 6	“Japan” (“日本”)	“COVID-19 Vaccine” (“COVID-19ワクチン”)
Top 7	“Germany” (“ドイツ”)	“Socioeconomic impact of coronavirus infections in 2019” (“2019年コロナウイルス感染症による社会・経済的影響”)
Top 8	“City of Yokohama” (“横浜市”)	“Amabile” (“アマビエ”)
Top 9	“September 11th attacks” (“アメリカ同時多発テロ事件”)	“The three Cs: closed spaces, crowded places, and close-contact settings” (“3つの密”)
Top 10	“Tokyo” (“東京都”)	“Declaration of an emergency and priority measures to prevent the spread of the disease” (“緊急事態宣言及びまん延防止等重点措置”)

The categories of the articles found in the editing histories of the 2 groups’ editors were investigated. For the social-political group, 55.42% (453,381/818,082) of the edited articles in their editing history were categorized as “Social” or “Political” articles, and only 1.22% (1996/163,606) was categorized as “Science” or “Medicine.” In contrast, for the scientific-medical group, only 3.24% (26,506/818,086) of the articles in their editing history were in the “Social” or “Political” categories, whereas 66.39% (108,625/163,616) were categorized as “Science” or “Medicine.”

Thus, COVID-19 information on Japanese Wikipedia was produced by a collaboration between 2 groups of editors, one with a strong preference for and knowledge of social and political topics and the other with a strong preference for and knowledge of science and medicine.

### A Leading Role of Editors With a Sociopolitical Preference in the Production of Articles on COVID-19 on Japanese Wikipedia

The extent to which the 2 groups of focal editors contributed to the production of COVID-19 information on Japanese Wikipedia was investigated.

First, with 1014 editors, the social-political group was much larger than the scientific-medical group, which only had 309 editors. This indicates that 76.64% (1014/1323) of the editors in producing COVID-19 information on Wikipedia strongly preferred social and political issues. In contrast, only 23.36% (309/1323) of these editors preferred scientific and medical topics.

The social-political group contributed 72.09% (23,003/31,908) of the edits of COVID-19 articles, and the scientific-medical group contributed 27.91% (ie, 8907/31,908 edits) of the edits of the articles on COVID-19.

Next, the social-political group contributed to 70.04% (16,544,495/23,485,683) of the bits changed on COVID-19 articles, whereas the scientific-medical group only contributed 29.06% (6,941,188/23,485,683) of the bits changed.

Finally, 75.61% (57,969/76,673) of the references were added by the social-political group, whereas 24.39% (18,704/76,673) of the references were added by the scientific-medical group.

To verify the robustness of the results, we repeated the abovementioned analyses by only using the data from the first few weeks and months. In addition, we examined the robustness of our results by considering the protection periods of the articles, that is, when the articles were locked for editing. The details can be found in Multimedia Appendix 1. To summarize, the additional analysis results confirmed that the social-political group played a central role in the information production process.

Furthermore, we also repeated the abovementioned static analyses using the data from February 2020 to April 30, 2020, and the results are provided in Multimedia Appendix 1. During this period, the first wave of the pandemic began to hit Japan, and the scientific-medical group and the social-political group contributed particularly more edits. However, even during this period, the social-political group was found to play a central role in the information production process.

**Figure 3:**
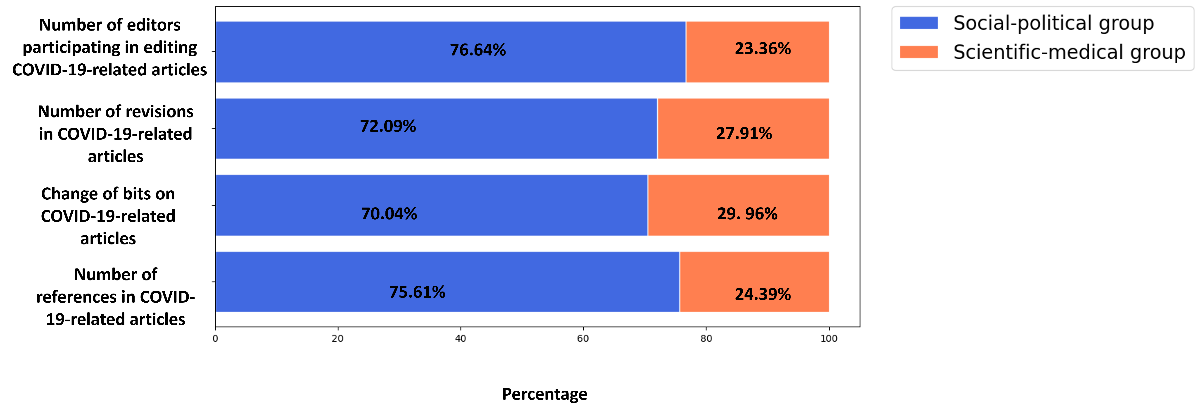
Summary of contributions of the social-political and scientific-medical groups to the information production of COVID-19 on Japanese Wikipedia. This figure presents (1) the ratio of editors participating in the COVID-19 information production on Wikipedia in the social-political group and the scientific-medical group, (2) the ratio of edits on COVID-19 articles implemented by the social-political and scientific-medical groups, (3) the ratio in the changes of bits on COVID-19 articles implemented by the social-political group and the scientific-medical group, and (4) the ratio of references in COVID-19 articles added by the social-political and the scientific-medical groups. The blue portion of the bars show the percentages contributed by the social-political group, whereas the orange bars show the percentage contributed by the scientific-medical group.

These results, shown in Figure 3, indicate that although COVID-19 is a medical or scientific matter [19], editors with a strong preference for sociopolitical topics played a leading role in producing COVID-19 information on Japanese Wikipedia.

### The Social-Political Group Played a Greater Role as the COVID-19 Pandemic Intensified

Finally, dynamic analyses were conducted. First, the results showed that from February 6 to 12, 2022, the contribution variable of the social-political group was >0.5 for 97.14% of this period. Thus, >97% of the time during this period, the social-political group played a predominant role in producing COVID-19 information on Wikipedia. In contrast, the scientific-medical group played a leading role in producing information about COVID-19 on Wikipedia <3% of the time.

A significant positive Pearson correlation was found between the contribution variable for the social-political group and the case variable (coefficient=0.23; *P*<.001). This indicates that the social-political group tended to play a more important role during higher levels of the COVID-19 pandemic in Japan (ie, larger numbers of new cases were identified in Japan). The scientific-medical group, for its part, tended to play a more prominent role as the COVID-19 pandemic ebbed (ie, fewer new cases were detected in Japan).

Finally, the Granger causality test showed that the dynamic change in the contributing variables of the 2 groups was caused by dynamic changes in the case variable (*F*_3,722_=4.35; *P*=.03). Thus, the increased severity in the COVID-19 pandemic activated the editing behaviors of the social-political group, stimulating them to make more contributions to COVID-19 information. Conversely, the increased severity of the pandemic inactivated the editing behaviors of the scientific-medical group, causing them to make fewer contributions to COVID-19 information.

These results were computed in time windows of 7 days. To examine the robustness of the abovementioned results, the analyses were repeated with time windows of 1 day, 3 days, and 10 days. As shown in Table 2, all results were consistent: >90% of the time, the social-political group played the leading role in COVID-19 information production on Wikipedia; in addition, the increased severity of the pandemic in Japan activated the editing behaviors of the social-political group but inactivated the editing behaviors of the scientific-medical group.

**Table 2:** Results of the contribution variable of the social-political group based on different moving time windows.

Value of the moving time window	Time where the social-political group’s contribution variable was >0.5 (%)	Pearson correlation between the social-political group’s contribution variable and the case variable	*P* value for the correlation	*F* value for the Granger causality test (*df*)	*P* value for the Granger causality test
1 day	93.2	0.177	.001	7.904 (3,730)	.005
3 days	97.1	0.231	.001	4.349 (3,726)	.03
7 days	97.1	0.180	.001	3.989 (3,722)	.047
10 days	98.2	0.310	<.001	2.172 (3,719)	.09

## Discussion

### Principal Findings

To summarize, the abovementioned results indicated that 2 main groups of editors were involved in producing COVID-19 information on Wikipedia. Furthermore, one group had a stronger knowledge preference for social-political topics, whereas the other group had a greater preference for scientific-medical topics. In addition, the social-political group played a predominant role in COVID-19–related information production on Wikipedia and produced a larger share of information about COVID-19 on Wikipedia; in contrast, the scientific-medical group played a secondary role and produced a relatively small portion of COVID-19 information on Wikipedia. Finally, increased severity of the COVID-19 pandemic in Japan activated the editing behaviors of the social-political group and was associated with more contributions to COVID-19 information; conversely, the same circumstance inactivated the editing behaviors of the scientific-medical group, resulting in fewer contributions to COVID-19–related information.

### Implications and Contributions

An important implication can be deduced from the principal results of this study. Previous research [43,44] pointed out that experts, especially scientists, tended to remain silent on issues with a high level of scientific uncertainty before the related information and data became available during media hypes (ie, in cases where the corresponding issues received exaggerated publicity in the mass media). The “silence of experts” during media hypes was considered an inevitable problem in the discussions of issues with high scientific uncertainty because experts essentially want to display their professionalism and expert knowledge by being highly accurate [43,44]. These research findings showed that the “silence of experts” is a more general phenomenon that also occurred in the process of knowledge coproduction of lay experts, who, in the science and medicine fields, tend to pursue scientific certainty and are concerned with producing accurate evidence. Consequently, they tended to remain silent when the corresponding issues (eg, COVID-19) received a large amount of attention from the public. However, the results of this research also imply that the absence of these lay experts in discussions in the first place is not necessarily a problem. Instead, as previous research about “postnormal science” [45] has suggested, the social and political context of the issues with high scientific uncertainty is more important than accuracy for discussions in the first place. In this respect, the results of the current research practically suggest that when dealing with future pandemics, the focus of the policies and countermeasures should be on understanding the social and political context as well as the scientific uncertainty of medical knowledge, especially in the early stage [45]. Only after a solid understanding of the social and political context is built should the focus be on the accuracy and certainty of the medical knowledge. Of course, these perspectives rely on the presumed objective correctness of the Wikipedia articles. However, this study could be evidence that specialized knowledge, which should be scientific, is subject to excessive sociopolitical bias. It is noteworthy that the results of this analysis of the process through which lay experts’ knowledge were developed in the Japanese context. In addition, the editing process is also highly dependent on the sociopolitical context for the formation of epistemic authority [46].

In addition to the abovementioned main contribution, the clustering methods of editors proposed in this research can contribute to future research to address similar issues with large-scale data. In previous research [47,48], methods based on the topic model were often used to cluster editors in terms of their history of edits. Compared with our method, the topic model requires the content of every article that the editors revised and is much more time consuming for training. In this sense, our method permits conducting clustering without processing text data. Therefore, it is faster and more flexible, which is especially suitable for large data sets.

### Limitations

Finally, it is worth noting that this study had certain limitations. First, it only focused on Japanese Wikipedia and the production of information about COVID-19. Future research should examine whether the results of this study are consistent with those of Wikipedia in other languages. Second, because of the inaccessibility of data, this research did not consider the demographic features of the editors (eg, gender, age, or occupation) into account. Previous research [16] found that these individual features can also primarily affect the information production behaviors of Wikipedia editors. Furthermore, we did not have space to discuss the epistemological controversy among the editors that might have caused a tumult in the Userbox [49,50]. Finally, by combining survey data with the data sets used in this study, future work can expand the analyses of this study to further discuss related issues.

Furthermore, although the results of this study are accepted, there remains the question of why there was partisan confusion in the editing process of COVID-19 articles on general Wikipedia in previous studies [26-28], whereas there was no such confusion on Japanese Wikipedia. There are also questions regarding why the change in roles in the editorial group was successful. One possible explanation is that the social positioning of science in the Japanese-speaking world is also a factor. For example, the compliant Japanese public reaction to the COVID-19 policy and our recent survey indicate that Japanese society may blindly trust science. It is possible that this cultural context also influenced the editing of Wikipedia. That is, editors in the social-political group may have trusted that editors in the scientific-medical group would ensure scientific accuracy and thus could conduct their editing work without hesitation. Although the impact of these social and cultural factors on the editing process is beyond the scope of this study, the results may provide an opportunity to consider how citizens engage in coproduction of scientific knowledge socially.

## Appendix

Multimedia Appendix 1 All supplementary analyses and results mentioned in the manuscript in 4 parts. The first part provides a quality analysis of the COVID-19–related Wikipedia articles. The second and third parts verified the robustness of the results in the manuscripts using different methods and periods of data. The final part provided information about the code and data availability of this research.

## Abbreviations

BIC: Bayesian information criterion
TF-IDF: term frequency–inverse document frequency
WHO: World Health Organization
