# Supplementary material for: Crowdsourcing Knowledge Production of COVID-19 Information on Japanese Wikipedia in the Face of Uncertainty: Empirical Analysis
Source: J Med Internet Res. 2023 Jun 29;25:e45024. doi: 10.2196/45024 (PMC10365582; doi:10.2196/45024)
Supplement: Multimedia Appendix 1 [file jmir_v25i1e45024_app1.pdf]

Appendix

Crowd-sourcing knowledge production of COVID-19 in the face of uncertainty: An empirical analysis of information on Japanese Wikipedia

This PDF file includes the following:

Supplementary text

Figures S1 to S14

Tables S1 to S6

S1. The high quality of COVID-19-related articles in Japanese Wikipedia

The COVID-19 information found on Japanese Wikipedia received an exceptionally high level of interest (e.g., over 12 million page views and 31,910 times of edits) relative to Wikipedia articles in other languages that are mainly spoken in a single country (e.g., Dutch, Korean). In this section, we performed the following analysis to verify the high quality of COVID-19 information in Japanese Wikipedia.

For English Wikipedia, the Wikimedia Foundation officially released a machine learning model that allows researchers to evaluate article quality in a unified way. However, there is no such tool to evaluate the quality of articles in Japanese Wikipedia. Therefore, based on previous research [48], we employed three indicators to measure the quality of 133 articles related to COVID-19 analyzed in the current research.

1). The number of edits: previous research [49] has suggested that the open edit system is a kind of peer review system in which editors' review and revise articles. As a result, an article that receives many edits can be considered verified through several rounds of peer review and, therefore, is expected to have a higher quality.

2). The number of pageviews: this reflects how many times readers access an article. Previous research [48] found that the number of pageviews can be considered an indicator of high quality articles, reflecting the article quality based on two mechanisms [49]. First, articles that provide valuable information are more likely to receive more pageviews. Second, more editors would participate in refining a featured article than an article that is not viewed as much.

3). The number of references: as explained in the “Methods” section of the manuscript, the number of references reflects the reliability of the content of an article, with a large number of references indicating higher reliability.

Based on the above three indicators, we measured the quality of 133 articles on COVID-19. We first gathered data from “excellent” articles (https://ja.wikipedia.org/wiki/Wikipedia: 秀逸な記事) and “good” articles (https://ja.wikipedia.org/wiki/Wikipedia: 良質な記事) in the field of medicine in Japanese Wikipedia as a control group. These articles were voted as having very high quality by editors in the Japanese Wikipedia based on strict criteria (only 0.007% of Japanese articles were voted as “excellent,” and 0.14% were voted as “good”). We then calculated the above three indicators and compared them between the control group and the article on COVID-19 articles. In Figure S1, we found that for all three indicators, there were no statistically significant differences between the control group and the COVID-19 articles. This result indicates that the quality of the articles on COVID-19 is as high as those whose quality has been verified by the editors.

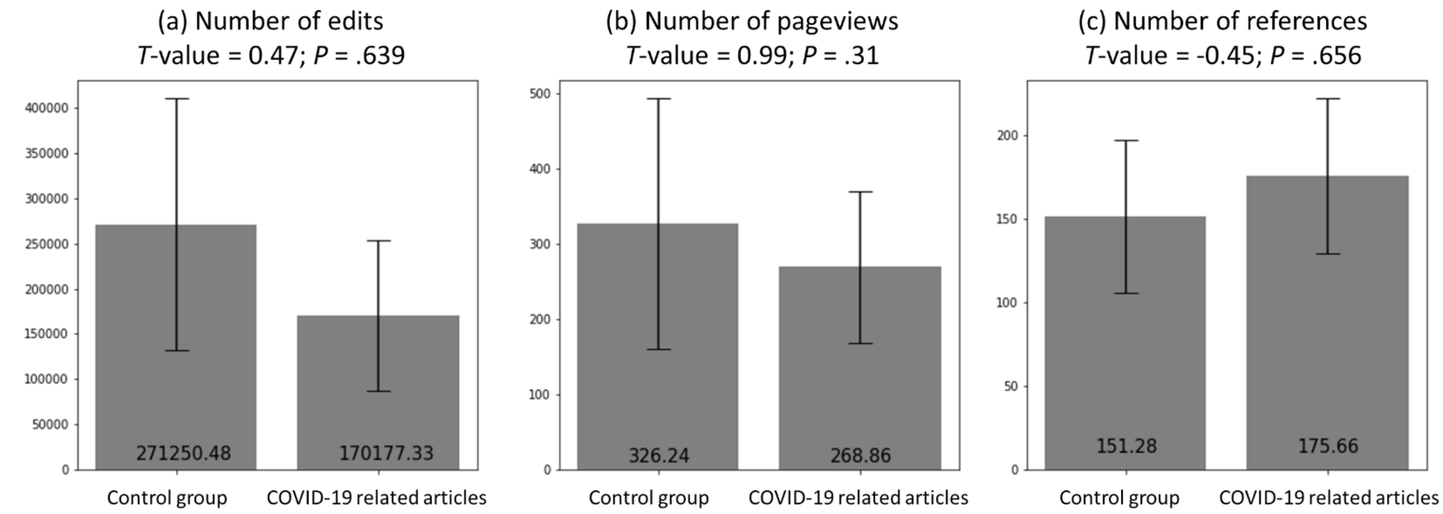

Figure S1. An illustration of the high quality of articles on COVID-19 in Japanese Wikipedia. The three panels show the average number of edits, the average number of pageviews, and the average number of references of the control group and the COVID-19 group, respectively. The error bars represent two times the standard errors of the means, and *T*-values and *P*-values were computed based on the *t-test*.

S2. The robustness of the results in the first wave of the pandemic.

In the manuscript, we analyzed all 31,910 edits of articles on COVID-19 from February 06, 2020 (i.e., the date when the first COVID-19 article was created in Japanese Wikipedia), to February 12, 2022 (i.e., the date when the data were collected). In this section, we verified whether our results were robust by considering 1) the anonymous editors' behavior, 2) the protection periods of the articles, and 3) the initial stage of the pandemic when the impact of COVID-19 on Japanese society was most significant.

First, we excluded the edits of 533 anonymous editors. These anonymous editors contributed 4,007 edits on the 133 articles on COVID-19, meaning 10.24% of all edits to these articles. Although this percentage may appear high, we found that the edits from anonymous editors barely changed the substantive content of the articles. It was found that the average bit change in the logarithm scale of anonymous editors (1.004) was roughly three times lower than that of registered editors (3.414). Additionally, anonymous editors only contributed 0.06% of references (10) that were cited by articles on COVID-19. Thus, it was suggested that instead of playing key roles (e.g., verifying the reliability of information by adding references, adding a new essential section to the articles), anonymous editors are mainly concerned with reformatting and nonessential aspects of the articles. We conclude that excluding anonymous editors did not undermine the robustness of the main conclusions in the manuscript.

Then, we further validated the robustness of our results by considering the protection periods of the COVID-19 related articles. Protection periods of a Wikipedia article is when the article's content is locked such that editors cannot make edits. Among the 133 articles on COVID-19, six were partially protected 21 times due to

vandalism. Each time, the articles were protected for a week to a year. A total of 4.20% of the edits (i.e., 1,339 edits) in our dataset were conducted when the articles were protected. Twice, the articles were only allowed to be edited by editors with an "extended-confirmed" access level who had been registered for at least 30 days and who had made more than 500 edits. In the remaining 20 times, "auto-confirmed" editors, who have been registered for at least 4 days and have made at least 10 edits, were allowed to edit the articles. The details of the 21 protections periods can be found in Table S1. In our dataset, 85.45% of the editors were auto-confirmed editors (including the extended-confirmed editors) who contributed 98.50% of the edits and 96.90% of the bit changes, while 40.8% of the editors were extended-confirmed editors who contributed 62.96% of the edits and 58.67% of the bit changes. Therefore, the influence of protection periods was considered minor. To further rule out the influence of these protections, we only used the editing histories of unprotected articles to reconduct our analyses. The results can be found in the last row of Table S2. All these results were consistent with those in the manuscript, showing that the soc-pol group played the leading role in the information production process.

Finally, to verify the robustness of our results during the initial stage of the pandemic, we repeated our analyses only using data from the first few weeks and months.

We considered five different periods: 1) the first week (i.e., from February 06, 2020 to February 13, 2020), 2) the first two weeks (i.e., from February 06, 2020 to February 20, 2020), 3) the first month (i.e., from February 6, 2020 to March 06, 2020), 4) the first six month (i.e., from February 06, 2020 to August 06, 2020), and 5) the first year (i.e., from February 06, 2020 to February 06 2021). In Table S1, it was found that all the results of the additional analysis were consistent with the results in the manuscript showing that the soc-pol group played a central role in the information production process. Additionally, to demonstrate these results more clearly, we drew three figures (see Figure S2) to show the cumulative ratios of the editing behaviors of the editors in the two groups. The cumulative ratio shows the percentage of edits, bit changes, or reference additions that have been conducted up to a certain day. For instance, if the editors added ten references across the whole observation period, and on the first day, they added one; on the second day, they added two. In this case, the cumulative ratio of reference addition was 10% (1/10) on the first day and 30% (1 + 2)/10) on the second day. By observing the slope of the cumulative ratio, we can determine whether a certain group of editors suddenly became active. We found that there was a period (roughly from February 2020 to April 2020) when the sci-med group added a particularly large amount of information (bits) and references to the articles. However, the soc-pol group also conducted many edits during this same period. We did not see a difference in the slopes of the cumulative ratios between the two groups. Since the first wave of the pandemic hit Japan during this period, we considered the exceptionally high activity of the two groups as a natural response to the pandemic. In addition to the cumulative ratio, we reconducted the same statistical analyses based on data from February 06, 2020 to April 30, 2020 (see details in Table S2).

The results did not change: the soc-pol group was still found to play the leading role during this period.

Table S1. Summary of protection periods for COVID-19 articles.

**Title of the articles**<br>**(*English translation*)**	**Date of the protection starting**	**Duration of the protection**	**If only allowed to be edited by extended editors**
SARS コロナウイルス 2<br>(*SARS coronavirus 2*)	2020/4/22	30	False
SARS コロナウイルス 2<br>(*SARS coronavirus 2*)	2020/6/1	183	False
新型コロナウイルス感染症の世界的流行 (2019 年-)<br>(*Global Pandemic of New Coronavirus Infections (2019-)*)	2020/8/18	31	False
新型コロナウイルス感染症の世界的流行 (2019 年-)<br>(*Global Pandemic of New Coronavirus Infections (2019-)*)	2020/11/4	14	False
新型コロナウイルス感染症の世界的流行 (2019 年-)<br>(*Global Pandemic of New Coronavirus Infections (2019-)*)	2020/12/15	90	False
3 つの密<br>(*Three Dense*)	2020/12/17	90	False
日本における 2019 年コロナウイルス感染症の流行状況<br>(*2019 Coronavirus Outbreak Status in Japan*)	2021/1/7	14	False
3 つの密<br>(*Three Dense*)	2021/3/28	31	False
3 つの密<br>(*Three Dense*)	2021/5/1	92	False
新型コロナウイルス感染症の世界的流行 (2019 年-)<br>(*Global Pandemic of New Coronavirus Infections (2019-)*)	2021/7/3	14	False
日本における 2019 年コロナウイルス感染症の流行状況<br>(*2019 Coronavirus Outbreak Status in Japan*)	2021/7/4	31	False
新型コロナウイルス感染症 (2019 年)<br>(*New Coronavirus Infections (2019)*)	2021/7/10	31	False
新型コロナウイルス感染症 (2019 年)<br>(*New Coronavirus Infections (2019)*)	2021/8/22	184	False
COVID-19 ワクチン<br>(*COVID-19 Vaccine*)	2021/8/30	92	True
MRNA-1273	2021/9/2	91	False

日本における 2019 年コロナウイルス感染症の流行状況<br>(*2019 Coronavirus Outbreak Status in Japan*)	2021/10/21	92	False
新型コロナウイルス感染症の世界的流行 (2019 年-)<br>(*Global Pandemic of New Coronavirus Infections (2019-)*)	2021/10/24	92	False
新型コロナウイルス感染症 (2019 年)<br>(*New Coronavirus Infections (2019)*)	2021/11/23	181	True
SARS コロナウイルス 2<br>(*SARS coronavirus 2*)	2021/11/28	92	False
3 つの密<br>(*Three Dense*)	2021/12/10	365	False
COVID-19 ワクチン<br>(*COVID-19 Vaccine*)	2022/1/9	90	False

Table S2. Summary of contributions of the soc-pol and sci-med groups to COVID-19 information on Japanese Wikipedia during different periods.

	Number (ratio) of editors in soc-pol group	Number (ratio) of edits in soc-pol group	Size (ratio) of bit change implemented by soc-pol group	Number (ratio) of references added by soc-pol group	The median (ratio) of bit change implemented by soc-pol group
**The first week**	38 (90.48%)	345 (77.70%)	101695 (89.80%)	19 (95.0%)	48.0 (1.66)
**The first two weeks**	67 (87.01%)	784 (77.93%)	319931 (89.12%)	67 (91.78%)	71.0 (3.74)
**The first month**	147 (81.67%)	2636 (68.83%)	2038518 (91.23%)	1004 (82.16%)	70.5 (4.70)
**First six months**	414 (77.24%)	6408 (57.52%)	6791059 (71.88%)	3009 (79.10%)	108.0 (3.38)
**The first year**	600 (78.53%)	9784 (61.31%)	9841938 (75.59%)	4127 (79.30%)	81.0 (2.31)
**Active period (6 February 2020 to 30 April 2020)**	754 (77.25%)	15941 (69.12%)	12623485 (71.32%)	4803 (77.06%)	23.0 (1.61)
**Without protection periods**	988 (77.31%)	21768 (71.20%)	16043089 (70.14%)	5527 (75.02%)	21.0 (1.55)

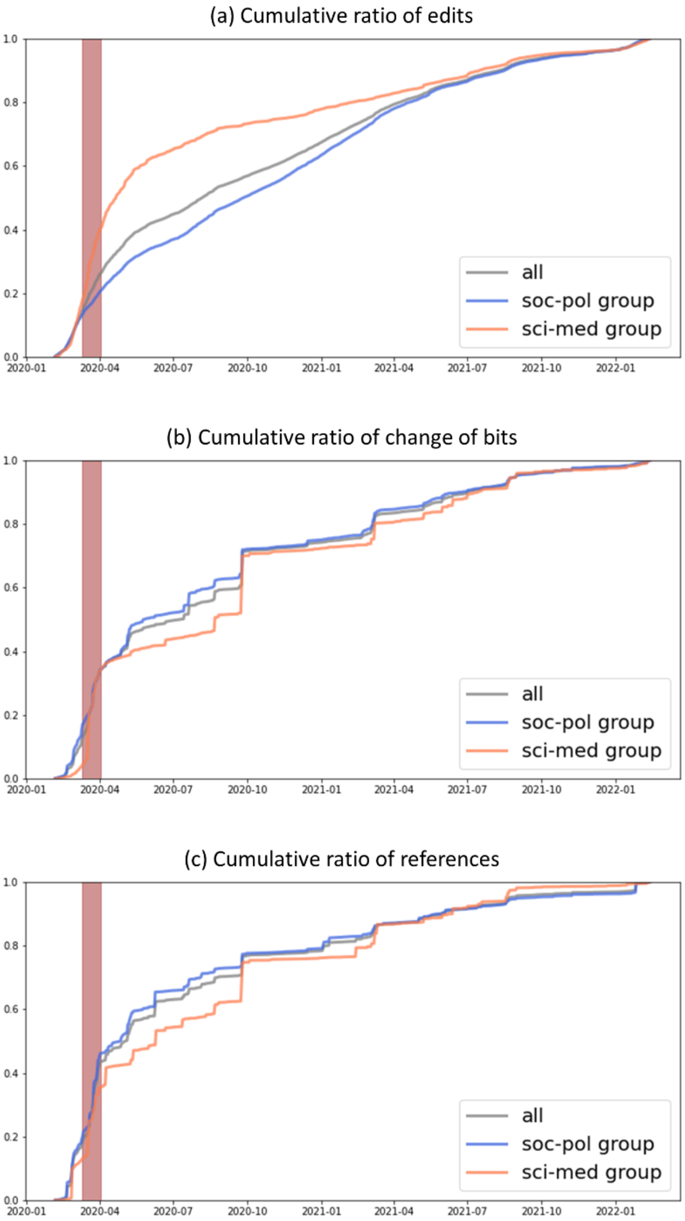

Figure S2. Illustration of the cumulative ratio of (a) the number of edits, (b) the number of bit changes, and (c) the number of references for the soc-pol group (in blue), the sci-med group (in red), and all editors (in grey). The red half-transparent bars demonstrate the period (roughly from February 2020 to April 2020) when the two groups became particularly active.

S3. Validation of the roles of the soc-pol and sci-med groups.

In the manuscript, the soc-pol group was found to make more edits, bit changes, and reference additions. Therefore, they were considered to play the main role in editing COVID-19 articles. However, an alternative explanation may be that the soc-pol group was in charge reformatting articles without providing essential information. To further rule out this alternative explanation, we addressed 1) the distribution of the medians of the number of articles for editors in these two groups, and 2) the distribution of the medians of the bit changes for editors in these two groups. If a group of editors mainly made reformatting changes instead of essential edits, the distributions of the medians should have a heavy body on the left of x-axis, reflecting that most of the editors only contributed to a small number of bit changes in a few articles. Figure S3 (a) shows the distribution of the medians of bit changes (in logarithm scale) for the editors in both the soc-pol sci-med groups. Figure S3 (b) shows the distribution of the number of articles (in logarithm scale) edited by the editors in the soc-pol and sci-med groups. The two figures indicate the same results: there was no evidence that either the soc-pol group or the sci-med group mainly performed reformatting. Instead, the results implied that the "role division" occurred within groups: both in the soc-pol group and the sci-med group, some of the editors were dedicated to reformatting (i.e., only changed the contents with few bits and only focused on one or two COVID-19 related articles), while the other editors produced essential content for the articles.

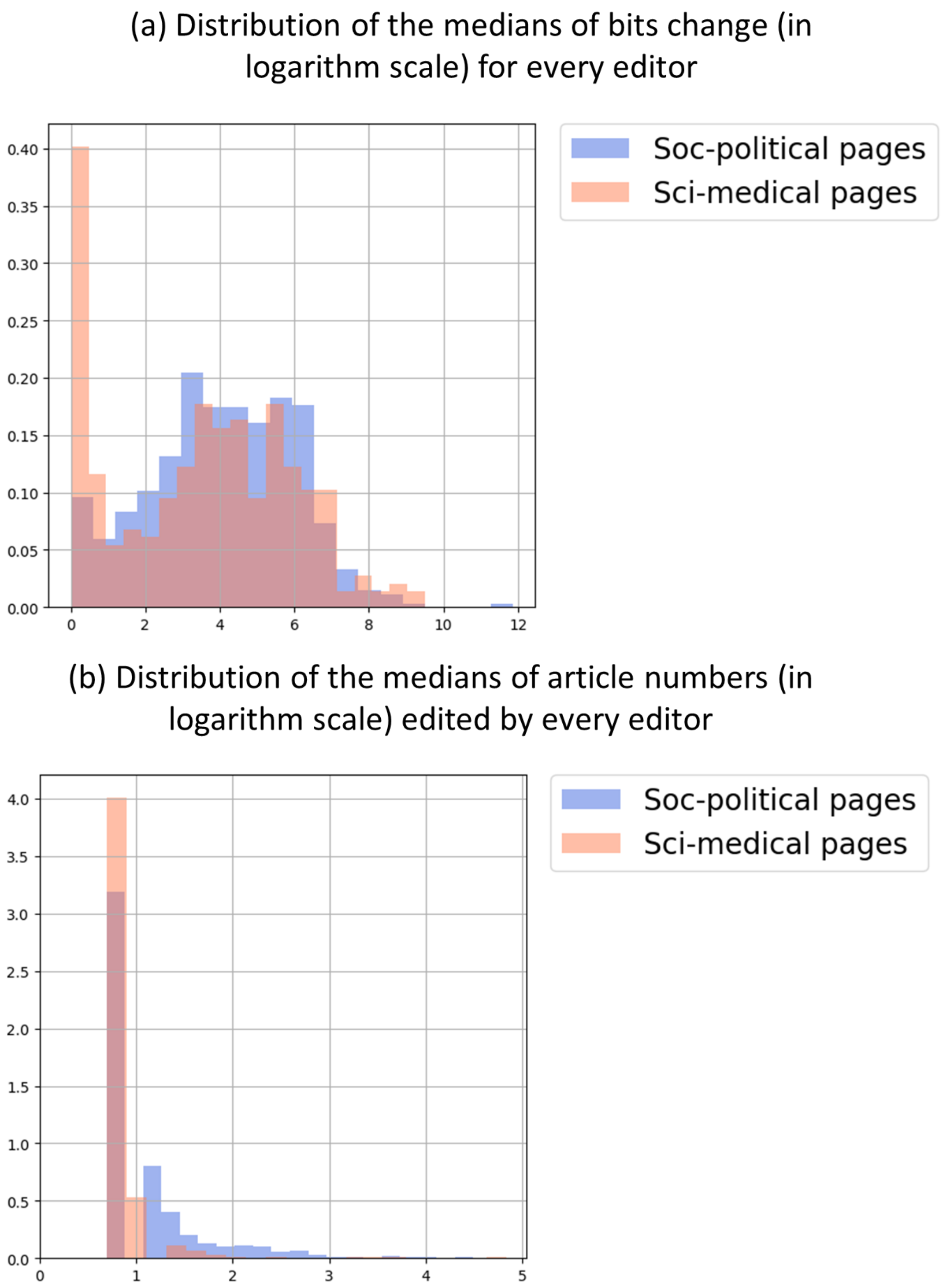

Figure S3. An illustration of the distributions of the medians of (a) the number of bit changes and (b) the number of articles edited by the soc-pol group (in blue) and the sci-med group (in red),.

S4 Code and data availability

The raw data and the Python code created for the statistical analyses, as well as the data for making the figures in the current study, are available in a dedicated OSF repository:

https://osf.io/yznd2/?view_only=b1fded185281422dbed6495ef923e4c8

We divided the code and the dataset into three parts: 1) a Wikipedia API crawler for collecting raw data in the same format as our analyses, which could help future researchers conduct similar analyses in various languages of Wikipedia articles; 2) the code and a pretrained model for replicating the results based on the raw data collected by the former crawler; and 3) a clean dataset and the corresponding code which allows interested readers to verify our results and explore our data based on their self-defined criteria. In particular, for the third part, we provided a predefined function (named “detector”), which allows readers with basic Python skills to freely explore the contributions of both groups of editors.
